# Liver and blood cytokine microenvironment in HCV patients is associated to liver fibrosis score: a proinflammatory cytokine ensemble orchestrated by TNF and tuned by IL-10

**DOI:** 10.1186/s12866-015-0610-6

**Published:** 2016-01-07

**Authors:** Soriane de Souza-Cruz, Marilú Barbieri Victória, Andréa Monteiro Tarragô, Allyson Guimarães da Costa, João Paulo Diniz Pimentel, Ericka Florêncio Pires, Lorene de Paula Araújo, Jordana Grazziela Coelho-dos-Reis, Matheus de Souza Gomes, Laurence Rodrigues Amaral, Andréa Teixeira-Carvalho, Olindo Assis Martins-Filho, Flamir da Silva Victória, Adriana Malheiro

**Affiliations:** Programa de Pós-Graduação em Imunologia Básica e Aplicada, Universidade Federal do Amazonas - UFAM, Manaus, AM Brazil; Departamento de Ensino e Pesquisa, Fundação de Hematologia e Hemoterapia do Amazonas - Hemoam, Manaus, AM Brazil; Programa de Pós-Graduação em Medicina Tropical, Universidade do Estado do Amazonas - UEA, Manaus, AM Brazil; Fundação de Medicina Tropical Doutor Heitor Vieira Dourado - FMT-HVD, Manaus, AM Brazil; Grupo Integrado de Pesquisas em Biomarcadores, Centro de Pesquisas René Rachou, Fundação Oswaldo Cruz, Belo Horizonte, MG Brazil; Laboratory of Biomarkers for Diagnosis and Monitoring, René Rachou Research Center – FIOCRUZ/MG, Av. Augusto de Lima 1715, Barro Preto, Belo Horizonte, CEP 30190-002 Minas Gerais Brazil; Laboratory of Bioinformatics and Molecular Analysis – INGEB / FACOM, Federal University of Uberlandia, Campus Patos de Minas, Major Jerônimo, 566, Lab 602, Patos de Minas, CEP 38700-002 MG Brazil

**Keywords:** Cytokine, Liver fibrosis, HCV

## Abstract

**Background:**

In this study, we have evaluated the immunological status of hepatitis C virus (HCV)-infected patients aiming at identifying putative biomarkers associated with distinct degrees of liver fibrosis. Peripheral blood and tissue T-cells as well as cytokine levels were quantified by flow cytometry.

**Results:**

Data analysis demonstrated higher frequency of circulating CD8^+^ T-cells and Tregs along with a mixed proinflammatory/IL-10-modulated cytokine pattern in HCV patients. Patients with severe liver fibrosis presented lower frequency of circulating CD8^+^ T-cells, higher levels of proinflammatory cytokines, but lower levels of IL-10, in addition to the higher viral load. Despite the lower frequency of intrahepatic T-cells and scarce frequency of Tregs, patients with severe liver fibrosis showed higher levels of proinflammatory cytokines (TNF and IFN-γ). The tissue proinflammatory cytokine pattern supported further studies of serum cytokines as relevant biomarkers associated with different liver fibrosis scores. Serum cytokine signature showed that mild liver fibrosis is associated with higher IL-10 serum levels as compared to severe liver disease. There was a clear positive connection of IL-10 with the TNF node in patients with mild liver fibrosis, whereas there is an evident inverse correlation between IL-10 with all other cytokine nodes.

**Conclusions:**

These results suggest the absence of modulatory events in patients with severe liver damage as opposed to mild fibrosis. Machine-learning data mining pointed out TNF and IL-10 as major attributes to differentiate HCV patients from non-infected individuals with highest performance. In conclusion, our findings demonstrated that HCV infection triggers a local and systemic cytokine ensemble orchestrated by TNF and tuned by IL-10 in such a manner that mirrors the liver fibrosis score, which highly suggests the relevance of these set of biomarkers for clinical investigations.

**Electronic supplementary material:**

The online version of this article (doi:10.1186/s12866-015-0610-6) contains supplementary material, which is available to authorized users.

## Background

Hepatitis C vírus (HCV) infects 130–170 million people worldwide, representing a global health problem [1–3]. Approximately 12–25 % of infected patients clear the virus spontaneously. However, the majority of HCV-infected patients remains infected and may evolve to the chronic phase of the disease, characterizing a silent epidemic [4]. The major complications of HCV infection are the progression to fibrosis, cirrhosis and hepatocellular carcinoma. These complications are related to high rates of morbidity and mortality [5, 6].

**Table 1:** Study population

Characteristics		Non-infected Controls (*n* = 28)	HCV Patients (*n* = 18)
Age		40.3 ± 9.1	50 ± 8.9
Male/Female		20/8	10/08
Liver Fibrosis Score	F1-2	NA	11
	F3-4	NA	7

The immune response in the HCV infection is responsible for partial viral elimination and protective immunity, but may also contribute to the liver damage [7]. HCV-induced liver damage is related to the robust tissue inflammation and hepatocytes destruction, which may result in progression to fibrosis. It has already been described that these factors are closely associated with cytokine production [8]. Cytokines are secreted upon HCV-mediated activation of immune cells and exhibit essential pleiotropical and adaptable functions in the immune response during the course of infection and liver damage [9]. Studies have shown that in chronic HCV infection there is a correlation between the production of proinflammatory cytokines, such as IFN-γ and TNF-α, and progressive liver injury, while the regulatory cytokines such as IL-4 and IL-10 may modulate the proinflammatory immune response induced by the virus, allowing for the establishment of a milder disease outcome [10]. The liver fibrosis has important implications for the prognosis of disease and for the decision-making on the onset of therapeutic approaches. The information about the state of fibrosis not only indicate response to treatment but also reflects and suggests the development of major complications such as liver cirrhosis [11, 12]. As of now, there is no clear knowledge of the relationship between the local and systemic production of cytokines and the participation of immune cells in liver fibrosis. Considering the lack of information on the local and systemic immunological biomarkers that correlate with the progression to liver fibrosis, this study aimed at investigating the balance between inflammatory and regulatory events in the peripheral blood and in the liver and their association with fibrosis score.

## Methods

### Study population

This study was approved by the Ethics Committee at the Tropical Medicine Foundation “Dr. Heitor Vieira Dourado” (Protocol#CEP:2339) and are in accordance with the Resolution 466/12 from the Brazilian Ministry of Healthy and also with the Helsinki Declaration of 1975. Signed informed consent was obtained from each participant for the use of biological materials and publication of data.

This study included 46 subjects living in Manaus, Amazonas, Brazil. Subjects were categorized into two subgroups referred as: Hepatitis C Virus-infected patients (HCV) infected patients and Non-infected patients (NI). HCV group comprised 18 untreated patients from both genders (10 males and 8 females), with age ranging from 26 and 66 years (50 ± 8.9) contacted at the Tropical Medicine Foundation Dr. Heitor Vieira Dourado (FMT-HVD), with positive serologic test for HCV infection (anti-HCV IgG detected by ELISA). Patients co-infected with hepatitis A, B, D, E, human immunodeficiency virus (HIV) and those who had started treatment for HCV infection or presented decompensated cirrhosis (Child-Pugh B or C) were excluded from the study. HCV patients were further sub-categorized into two subgroups referred as F1-2 (n = 11) and F3-4 (n = 7) to according the METAVIR score for liver fibrosis (Table 1).

The control group was composed of 28 healthy subjects from both genders (20 males and 8 females), with age ranging from 25 and 58 years (40.3 ± 9.1) (Table 1), recruited as blood donor candidates at “Hospital Foundation of Hematology and Hemotherapy of Amazon (FHEMOAM) with negative results for serologic screening tests, including viral hepatitis (A, B and C), HIV, syphilis, Chagas disease and HTLV-1/2.

### Biological samples

Five milliliters of whole blood were collected in EDTA tubes from each patient to perform the phenotypic analysis of circulating leukocytes by flow cytometry. An additional 10 mL of blood was drawn into tubes without anticoagulant to obtain serum used to quantify the levels of circulating cytokines by cytometric beads array. Fine-needle liver biopsies were collected from a subgroup of HCV patient to obtain tissue specimens used for histological, immunophenotypic and cytokine analyses.

### HCV viral load analysis

HCV genomic RNA test (HCV-RNA) was performed in the Amplicor RT-PCR (Roche, NJ, USA), that presents a sensitivity of 50 IU/mL. Samples with detectable HCV-RNA were further genotyped throughout in-house RT-nested PCR and RFLP analysis [13, 14] and viral load determined by RT-PCR (Amplicor HCV Monitor, Roche, NJ, USA) with data expressed as IU/mL.

### Serum Alanine Transaminase (ALT) measurement

ALT activity was determined in serum samples collected by venous puncture by using the ALT test (Abbott Laboratories, Chicago, IL) and data was reported as international units (IU)/L.

### Histological analysis

Fine needle liver biopsies were processed by the Central Pathology Laboratory at FMT-HVD. Sections (4–5 μm) of formalin-fixed and paraffin-embedded liver specimen were stained with hematoxylin-eosin, reticulin and trichrome dyes. The METAVIR score was used to classify the liver fibrosis as either F1-2 or F3-4.

### Preparation of intrahepatic leukocytes and tissue homogenates

Fine needle biopsies were homogenized in tissue grinder to obtain single cell suspension and tissue homogenate for soluble cytokine analysis. Briefly, one section of the fine needle biopsy was immersed in 1 mL of RPMI and macerated using a pestle. The cell suspension was centrifuged to obtain the supernatant used for tissue cytokine quantification. The pellet was resuspended in phosphate-buffered saline (PBS) supplemented with 5 % bovine serum albumin and used for immunophenotypic analysis.

### Immunophenotypic analysis of whole blood leukocytes

An aliquot of 100 μL of EDTA whole blood or 50 μL of tissue cell suspension was incubated in the presence fluorescent labeled anti-human cell surface monoclonal antibodies (anti-CD3-PerCP/CloneSK7, anti-CD4-PE/Clone RPA-T4, anti-CD8-FITC/CloneHIT8a, anti-CD25-APC/CloneM-A251, anti-FoxP3-PE/Clone259D/C7, all purchased from BD Bioscience, San Diego, CA, USA) to identify helper and cytotoxic T-cell subsets and Tregs. Following incubation, cells were treated with 1 mL of erythrocyte lysing solution for 10 min at room temperature. After one wash step with PBS, cells were fixed in MFF fix solution (10 g/L of paraformaldehyde, 10,2 g/L of sodium cacodilate and 6.63 g/L of sodium chloride, pH7.2). Stained cells were stored at 4 °C up to 24 h prior flow cytometric acquisition. A total of 10,000/100,000 events were acquired for each blood or tissue samples to quantify T-cell subsets and Tregs, respectively. A dual laser FACScalibur flow cytometer (488 nm and 633 nm) was used for data acquisition and storage as FCS files. The FlowJo software (TreeStar Inc., Ashland, OR, USA) was used for data analysis. The results were expressed as percentage of positive cells within the lymphocyte gate.

### Cytokine analysis

The serum cytokines quantification (IL-6, TNF, IL-2, IFN-γ, IL-4, IL-10 and IL-17) was performed by Cytometric Bead Array (CBA kit, BD Biosciences Pharmingen, USA) according to the manufacturer’s specifications. A dual laser FACScalibur flow cytometer (488 nm and 633 nm) was used for data acquisition and storage as FCS files. The FCAP array software was used for data analysis using the fourth logistic parameter regression to calculate the results. Data was expressed as mean fluorescence intensity (MIF) for each serum or tissue cytokine.

### Data mining and statistical analysis

#### Conventional statistics

Comparative analysis between groups were performed to evaluate the frequency of peripheral blood or tissue T-cell subsets and Tregs as well as the tissue or serum cytokines between groups, using the GraphPad Prism software, version 5.0 (San Diego, CA, USA) using the nonparametric Mann-Whitney test. Significance were considered in all cases at *P* < 0.05.

#### Serum cytokine signature analysis

The serum cytokine signature analysis was carried out according to Luiza-Silva et al. [15]. This innovative model was designed to convert quantitative cytokine measurements in a categorical analysis of low and high cytokine producers. Briefly, the frequency of subjects with “High” serum cytokine levels were determined taking the global median value of serum cytokine levels obtained from the whole data set (HCV patients + NI Controls) as described [15]. The following values were used as the cut-off to segregate low and high cytokine producers (IL-6 = 137.3; TNF = 81.5, IL-2 = 121.5; IFN-γ = 56.5; IL-4 = 129.4; IL-10 = 95.1 and IL-17 = 60.1). Ascendant tissue cytokine signatures were assembled for each clinical group. The differences of cytokine signatures between groups were addressed by overlaid cytokine hierarchical curves, considering as “relevant” frequencies of high cytokine producers those groups with frequencies above the 50th percentile.

#### Biomarkers networks assembling

Biomarker networks were assembled to assess the association amongst serum cytokines. The correlations were significant when Spearman’s test resulted in a *P* < 0.05. Significant correlations were compiled using the open-access software Cytoscape (version 3.1.1), as previously reported [16, 17]. The biomarker networks were constructed using circular nodes for each cytokine including proinflammatory IL-6, TNF, IL-2 and IFN-γ (black circles), modulatory IL-10 axis (light gray circle) and additional IL-4 and IL-17 axes (dark gray circles). Connecting edges represent correlation scores categorized as positive strong (r ≥ 0.68; thick continuous line), positive moderate (0.36 ≥ r ≤ 0.67; thin continuous line), negative strong (r ≤ −0.68; thick dashed line), negative moderate (−0.36 ≥ r ≤ −0.67; thin dashed line) as proposed by Taylor [18].

#### Heatmaps analysis

The heatmaps were produced using the heatmap.2 function in the R (Project for Statistical Computing Version 3.0.1) and gplots package. Decision trees to select the minimal set of immunological features that efficiently segregated groups were built using the WEKA software (Waikato Environment for Knowledge Analysis, version 3.6.11, University of Waikato, New Zealand). The classification accuracy of the decision trees when applied to new data sets with unknown class labels was further evaluated by a 10-fold cross validation methodology available in the WEKA software.

## Results

### HCV patients with severe liver fibrosis (F3-4) presented higher viral load as compared to those with mild fibrosis

**Fig. 1:**
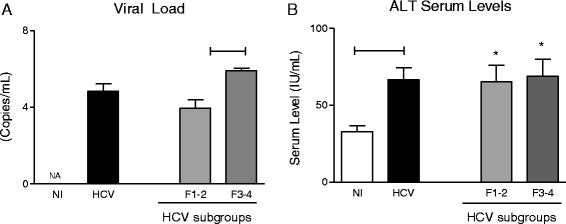
Virological and liver damage (ALT) assessment in HCV patients according to the fibrosis score. **a** HCV viral load and (**b**) serum levels of alanine aminotransferase (ALT) were determined in serum HCV patients () categorized into subgroups referred as F1-2 () and F3-4 () and compared to uninfected controls (). The results are expressed as mean ± SE viral copies/mL and mean ± SE IU/L, respectively. Significant differences at *P* < 0.05 between HCV versus NI or F1-2 versus F3-4 are highlighted by connecting lines. Differences between HCV subgroups (F1-2 or F3-4) in comparison to NI are highlighted by “*”. NA = Non Applicable

Aiming to characterize the virological and liver damage status in HCV patients according to the fibrosis score, we have evaluated the viral load and the ALT levels serum levels which are presented in Fig. 1. Data analysis demonstrated that HCV patients with severe liver fibrosis (F3-4) presented higher viral load as compared to those with mild fibrosis (Fig. 1). The ALT data revealed that HCV patients presented high levels of this liver damage biomarker but no significant differences between patients with mild or severe liver fibrosis were observed (Fig. 1).

### HCV patients display high frequency of CD8^+^ T-cells and Tregs along with a mixed proinflammatory/IL-10-modulated cytokine pattern

**Fig. 2:**
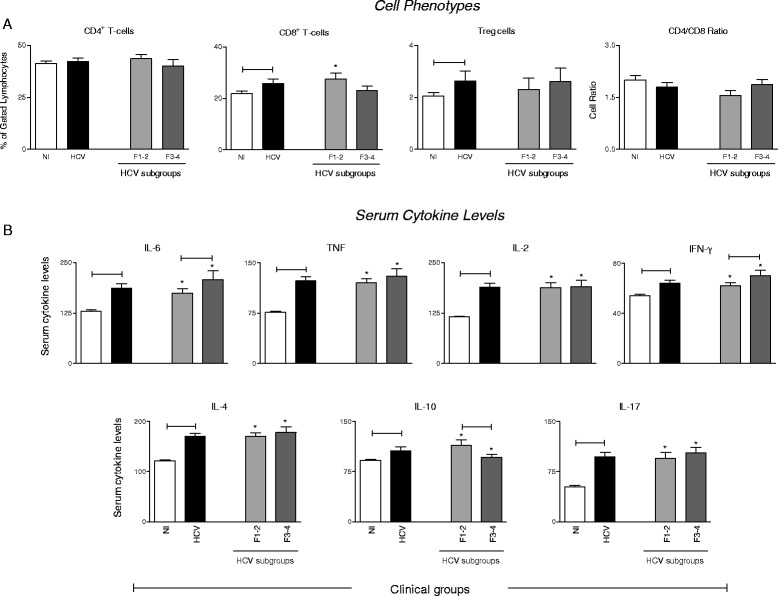
Peripheral Blood Biomarkers in HCV patients according to the fibrosis score. **a** Frequency of circulating T-cell subsets (CD4^+^, CD8^+^, Treg and CD4^+^/CD8^+^ ratio) in HCV patients () categorized into subgroups referred as F1-2 () and F3-4 () as compared to uninfected controls (). **b** Levels of serum cytokines (IL-6, TNF, IL-2, IFN-γ, IL-4, IL-10 and IL-17). Data are expressed as mean ± standard deviation for the percentage of gated lymphocytes for circulating T-cell subsets or serum concentration (MFI) for cytokines. Statistical analyses were performed by the Mann–Whitney test for comparisons between groups. Significant differences at *P* < 0.05 between HCV versus NI or F1-2 versus F3-4 are represented by connecting lines. Differences between HCV subgroups (F1-2 or F3-4) in comparison to NI are highlighted by “*”

In an attempt to determine whether peripheral blood immunological biomarkers in HCV patients associate to the liver fibrosis score, we have evaluated the frequency of major T-cell phenotypes that are presented in Fig. 2. Data analysis demonstrated that HCV patients display high frequency of CD8^+^ T-cells and Tregs (Fig. 2a) along with a mixed proinflammatory cytokine pattern (IL-6, TNF, IL-2, IFN-γ), modulated by IL-10 along with a prominent IL-4 and IL-17 production as compared to non-infected controls (Fig. 2b). No differences were observed neither in the frequency of CD4^+^ T-cells nor in the CD4^+^/CD8^+^ T-cell ratio (Fig. 2a).

### HCV patients with severe liver fibrosis (F3-4) show lower frequency of circulating CD8^+^ T-cells besides higher levels of proinflammatory cytokines and lower levels of IL-10

Further analysis of peripheral blood biomarkers including the quantitation of serum levels of proinflammatory and regulatory cytokines in HCV patients according to the fibrosis score are presented in Fig. 2. The results demonstrated that HCV patients with moderate fibrosis (F1-2) presented high frequency of CD8^+^ T-cells (Fig. 2a) along with lower levels of proinflammatory cytokines (IL-6 and IFN-γ) but higher IL-10 levels as compared to those patients with severe liver fibrosis (F3-4) (Fig. 2b).

### Despite the lower frequency of intrahepatic CD4^+^, CD8^+^ T-cells, HCV patients with severe liver fibrosis (F3-4) have higher levels of proinflammatory cytokines (TNF and IFN-γ) and the presence of scarce frequency of Tregs as compared to those with mild liver disease (F1-2)

**Fig. 3:**
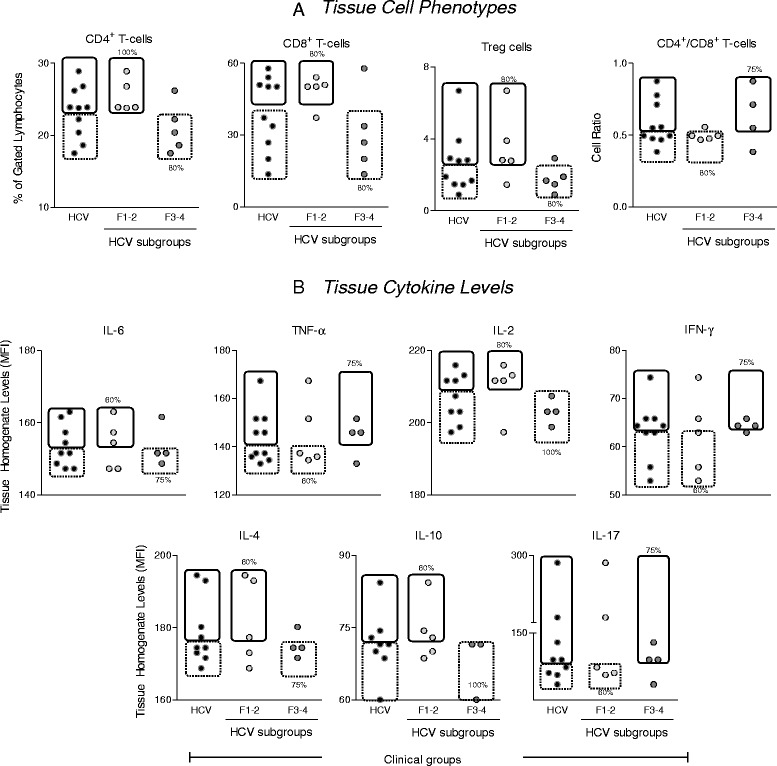
Hepatic Tissue Biomarkers in HCV patients according to the fibrosis score. **a** Frequency of intrahepatic T-cell subsets (CD4^+^, CD8^+^, Treg and CD4^+^/CD8^+^ ratio) in HCV patients () categorized into subgroups referred as F1-2 () and F3-4 (). **b** Levels of tissue cytokines (IL-6, TNF, IL-2, IFN-γ, IL-4, IL-10 and IL-17) secreted by mononuclear cells cultured in vitro for 12 h in RPMI (1x10^6^cells/mL) at 37 °C, 5 % CO_2_. Data are expressed as scattering of individual percentage of intra-hepatic T-cell subsets within gated lymphocytes or tissue for cytokine concentration (MFI). The median values of each biomarker was calculated for the HCV group and used as a cut off to segregate subjects presenting high (continuous lined rectangle) or low (dashed lined rectangle) biomarker levels. The Chi-square test was used to compare the frequency of subjects presenting high or low biomarker levels between the HCV subgroups (F1-2 versus F3-4). The frequencies of subjects presenting high or low biomarker levels in the F1-2 versus F3-4 subgroups are provided in the figure. In all cases, significant differences at *P* < 0.05 were found between F1-2 versus F3-4

Aiming to validate whether the observed pattern of peripheral blood biomarkers (Figs. 1 and 2) reflect the overall hepatic tissue immunological milieu, we have assessed the tissue cell phenotypes and tissue cytokine levels in HCV patients according to their liver fibrosis by flow cytometry, which are presented in Fig. 3. Data analysis demonstrated that although HCV patients with severe fibrosis (F3-4) presented low frequency of intrahepatic CD4^+^, CD8^+^ T-cells (Fig. 3a), they present higher tissue levels of proinflammatory cytokines (TNF and IFN-γ) along with low frequency of Tregs as compared to patients with mild liver fibrosis (F1-2) (Fig. 3a and b). Taken together, these data demonstrates that regardless the divergences in the frequency of intrahepatic T-cells, HCV patients have comparable tissue levels of proinflammatory cytokines (TNF and IFN-γ) according to their liver fibrosis score. The similarities between tissue and peripheral blood cytokine patterns supported the further use of serum cytokines as putative biomarkers associated to the liver fibrosis score.

### Cytokine signature analysis further highlight that HCV patients with mild liver fibrosis (F1-2) presented higher IL-10 serum levels as compared to those with severe liver disease (F3-4)

**Fig. 4:**
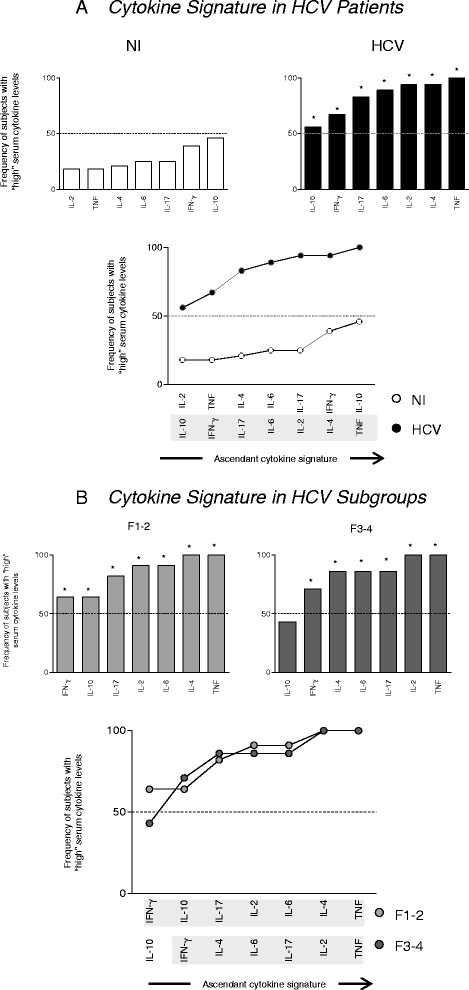
“Cytokine signatures” in HCV patients according to the fibrosis score. The ascendant frequency of subjects with “high” serum cytokine levels was assembled for (**a**) HCV patients () and uninfected controls () as well as (**b**) for the HCV patients categorized into subgroups referred as F1-2 () and F3-4 (). Relevant elements in the cytokine signature that emerge above the 50th percentile (cut-off line) were highlighted by *. Additional analyses were carried out to identify relevant elements in the cytokine signature able to differentiate clinical groups (HCV versus NI or F1-2 versus and F3-4) and the elements in the cytokine signature of each group that emerge above the 50th percentile were highlight by grayscale background rectangles

The analysis of cytokine signatures in HCV patients categorized according to their liver fibrosis score are shown in Fig. 4. Our data demonstrated that HCV patients presented a cytokine storm characterized by relevant frequency of high serum cytokine producers as compared to non-infected controls (Fig. 4a). The analysis of cytokine signatures further underscored that the subgroup of patients with mild liver fibrosis (F1-2) presented relevant frequency of subjects with high IL-10 serum levels as compared to patients with severe liver fibrosis (F3-4) (Fig. 4b).

### There is a clear positive connection of IL-10 controlling the TNF node in HCV patients with mild liver fibrosis (F1-2), whereas an evident negative axes of IL-10 with all other cytokine nodes highlights the absence of modulatory events in HCV patients with severe liver fibrosis (F3-4)

**Fig. 5:**
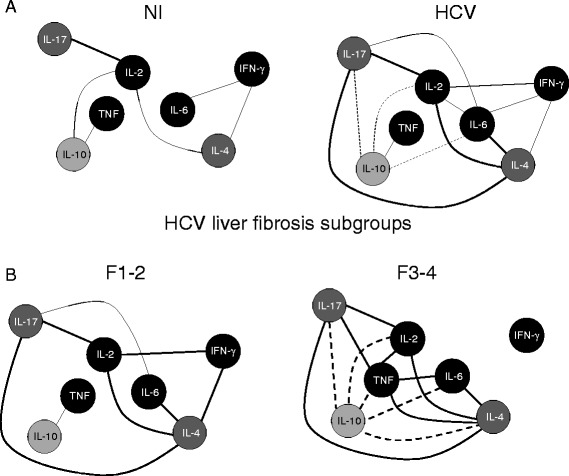
“Cytokine Networks” in HCV patients according to the fibrosis score. Customized biomarker network layouts were built to identify the relevant association between proinflammatory IL-6, TNF, IL-2 and IFN-γ cytokines (*black circles*), modulatory IL-10 axis (*light gray circle*) and additional IL-4 and IL-17 axes (*dark gray circles*), using a clustered distribution of nodes. Significant Spearmam’s correlations at *P* < 0.05 were represented by connecting edges to highlight positive [strong (r ≥ 0.68; thick continuous line) or moderate (0.36 ≥ r ≤ 0.67; thin continuous line)] and negative [strong (r ≤ −0.68; thick dashed line) or moderate (−0.36 ≥ r ≤ −0.67; thin dashed line)] as proposed by Taylor [18]. The overall statistic analysis of the network node neighborhood connections point out for an almost linear-chain pattern in the NI groups with a clear shift towards a more imbricate profile in HCV patients. A persistent IL17/IL-4 loop was observed in all HCV subgroups with differential neighborhood connections for the IL-10 node in HCV patients according to the fibrosis score

Data analysis demonstrate that HCV patients presented a more imbricate cytokine network amongst proinflammatory cytokines (continuous axes) counterbalanced by inverse relationship with IL-10 (dashed axes) and direct correlation between IL-4 and IL-17 axes (Fig. 5a). Regardless the liver fibrosis score, this direct correlation between IL-4 and IL-17 axes is preserved (Fig. 5b). HCV patients with mild liver fibrosis (F1-2) showed stronger connections between proinflammatory cytokines with a clear positive connection of IL-10 controlling the TNF node. On the other hand, HCV patients with severe liver fibrosis (F3-4) displayed significant inverse correlation between IL-10 and all other cytokines (Fig. 5b).

### Machine learning data mining pointed out TNF and IL-10 as the major attributes to identify HCV patients in comparison to non-infected individuals with outstanding performance

**Fig. 6:**
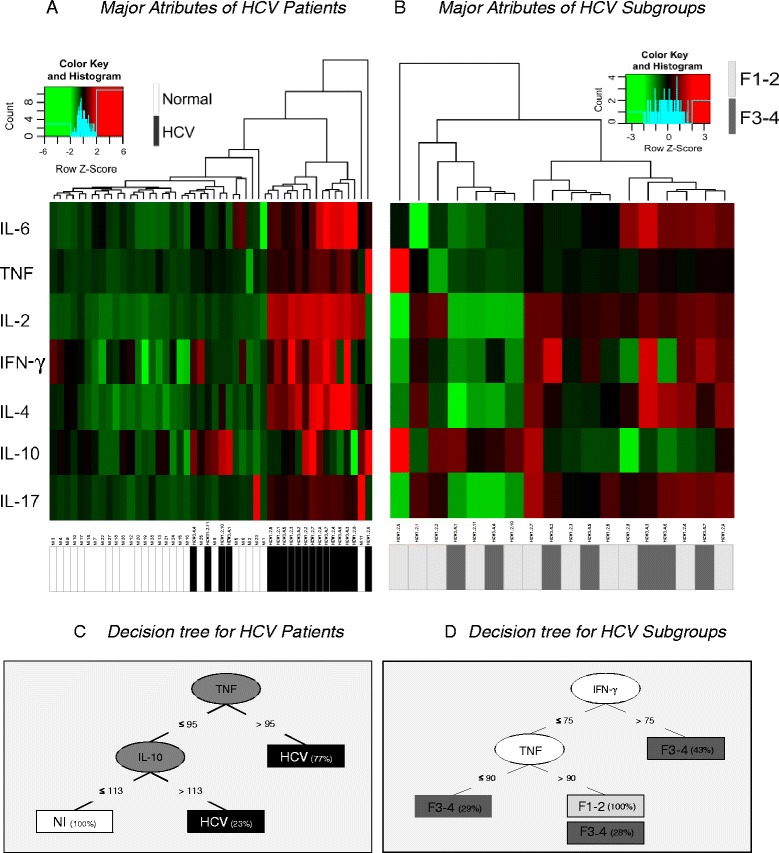
Computational bioinformatics analysis of serum cytokine levels in HCV patients according to the fibrosis score. Machine learning data mining was represented by heatmaps (**a** and **b**) and decision-trees (**c** and **d**) of z-score-normalized events. The heatmap computational method was applied to pre-process the serum cytokine data, in addition to identifying the attributes matching those across samples and clusters of individuals. **a** Serum cytokine attributes showed clear ability to cluster HCV patients, with up-regulated levels, apart from non-infected individuals, with basal levels of serum cytokines. **b** Heatmap analysis showed low performance to segregate the HCV patients according to their liver fibrosis scores. **c** Decision tree analysis provided the identification of “root” (TNF) and “secondary” (IL-10) attributes with high accuracy to categorize HCV patients aside from non-infected individuals. **d** Decision tree analysis showed moderate global performance of “root” (IFN-γ) and “secondary” (TNF) attributes to cluster HCV patients according to their liver fibrosis score

Computational bioinformatics analysis of serum cytokine levels in HCV patients clearly demonstrated the ability of these attributes to cluster HCV patients, with up-regulated levels apart from the basal levels observed in non-infected individuals (Fig. 6a). On the other hand, the heatmap analysis does not show the same performance to segregate the HCV patients according to their liver fibrosis scores (Fig. 6b). The decision trees further revealed that TNF and IL-10 are the major attributes able to categorize HCV patients aside from non-infected individuals with a global accuracy of 100 %, reaching 86 %, after leave-one-out cross validation (Fig. 6c). Trellis scatter plot for serum cytokine analysis reinforce the outstanding ability of the selected attributes to segregate the HCV patients from non-infected individuals, pointing out the relevant cytokine storm associated with HCV infection (Additional file 1: Figure S1). In contrast, the decision trees generated to cluster HCV patients according to their liver fibrosis score showed a global accuracy of 88.9 %, reaching 77.8 %, after leave-one-out cross validation (Fig. 6d).

## Discussion

HCV infection is a severe liver disease that constitutes a major health problem associated with high rates of morbidity and mortality due to recurrent complications, such as fibrosis progression, liver cirrhosis and hepatocellular carcinoma [5, 6]. The immune response against HCV infection has a pivotal but dual role during the course of infection. The early immunological events triggered by the virus have the potential to contribute to viral elimination and induce protective immunity, however, as the disease progresses; the late immunological events during the chronic phase may lead to liver damage [7].

The role of proinflammatory response is well discussed in the chronic phase of HCV infection and several studies have shown the importance of cytokines such as IL-1β, TNF, IFN-γ, IL-2, IL-6 and IL-12 during the course of HCV infection. It was reported variations in serum cytokine levels such as an increase in IFN-γ and IL-2, IL-4 and the regulatory IL-10 in the serum from patients with HCV infection as compared to the control group [19, 20]. Other studies reported a decrease of IL-4 [21], while Zhang et al. [22] reported a decrease in IL-2 serum levels of HCV patients. Previous data from our group have demonstrated the importance of TNF in HCV infection and treatment along with other proinflammatory cytokines such as IL-12, IFN-γ, IL-6, as well as the regulatory cytokines such as IL-4 and IL-10 [23].

Regarding the regulatory cytokines, studies demonstrated that the serum levels of IL-4 and IL-10 were significantly increased in the control group than the patients [24–26]. However, Sofian et al. [27] demonstrated that, both the concentration of the proinflammatory IL-2 and IFN-γ as well as the regulatory IL-4 and IL-10 are increased in serum of HCV patients as compared to the control group. In the analysis of serum cytokines in HCV patients, however, no significant differences were found in relation to the progression fibrosis between the groups ≤ F2 and ≥ F3. The stringency in categorizing patients according to their fibrosis score may vary, which may lead to divergence in results.

However, analyzing the individuals into “high” and “low” producers of cytokines in serum of control group, moderate fibrosis ≤ F2 and severe fibrosis ≥ F3 group showed that there is no high cytokine producers in the control group, while in the group of moderate fibrosis (≤F2) there is an elevated frequency of high cytokine producers with particularly high concentrations of IL-4, followed by TNF-α, IL-2, IL-6, IL-17, IFN-γ and IL-10. These data suggest an important role of IL-4 in controlling the development of hepatic lesions in the group of moderated fibrosis, whereas IL-2 could contribute to the development of severe fibrosis. The development of regulatory response is supported by IL-4, which antagonizes the production of IFN-γ and IL-2 promotes the development of a robust proinflammatory profile, which antagonizes the development of a regulatory environment [28]. Studies showed than the exchange of cytokines has been associated with increased activity necrosis followed by inflammation during liver fibrosis [29, 30].

The group of severe fibrosis ≥ F3 demonstrated a high percentage of individuals with particularly high concentrations of IL-2, followed by TNF-α, IL-4, IL-6, IL-17, IFN-γ. An interesting finding was that in the group with severe fibrosis ≥ F3, there is no relevant percentage of IL-10 high producers, which highly suggests that this cytokine may be involved in the control of inflammation-induced fibrosis formation. IL-10 is a regulatory cytokine that negatively regulates the expression of MHC-II and co-stimulatory molecules on antigen presenting cells, and inhibits T-cell activation via co-molecule stimulatory CD28 inhibition [31, 32]. The persistently elevated levels of IL-10 during HCV infection supposedly produced by suppressing regulatory T-cells may be an attempt to control fibrosis, thus contributing to the decline of the effector response against viruses and, therefore, preventing lesion expansion mediated by excessive cytotoxicity [33, 34, 35]. On the other hand, the low production of this cytokine may result in increased inflammatory responses and consequently excessive aggression to liver tissue.

Aroucha et al. [36] indicate a protective role of IL-10 in patients with moderate fibrosis, reinforcing our hypothesis that IL-10 plays a protective role in HCV infection regarding the progression of hepatic fibrosis. Other studies have emphasized the protective role of IL-10 used in the treatment of chronic hepatitis c infection, which had decreased severity of fibrosis in enrolled individuals [37]. In other studies with animal models, it was demonstrated that the absence of IL-10 was associated with liver fibrosis [38, 39]. In contrast to these data in a study by Abayli et al. [25] using IL-10 and histological activity, there was no correlation between these parameters.

In summary, the cytokine signature shows absence of high producers among HCV patients with moderate fibrosis (≤F2), which were characterized mainly by an increase of IL-4 and IL-10, while patients with severe fibrosis (≥F3) were characterized mainly by an increased IL-2 and decreased IL-10, reinforcing our hypothesis of the protective effect of the regulatory cytokines specially IL-10 in these subjects, as previously mentioned.

The activity of proinflammatory and regulatory cytokines is a dynamic event that will influence the progression of liver injury, contributing to reducing the damage caused by the virus or regulating the excessive response of effector cells. The knowledge about the mechanisms that control the production and secretion of such cytokines during the progression of liver fibrosis may contribute to the identification of potential therapeutic biomarkers to assess disease progression and alleviate the complications related to HCV infection.

## Conclusion

In conclusion, our findings demonstrated that HCV infection triggers a local and systemic cytokine ensemble orchestrated by TNF and tuned by IL-10 in such a manner that mirrors the liver fibrosis score, which highly suggests the relevance of these set of biomarkers for clinical investigations.

## Additional file

Additional file 1: Figure S1. Trellis scatter plot for serum cytokine analysis in HCV patients and non-infected controls. Scatter plot matrix analysis pointed out the relevant cytokine storm associated with HCV infection and reinforce the outstanding ability of serum cytokine attributes to cluster the HCV patients apart from non-infected individuals. (PPTX 2359 kb)

## Abbreviations

ALT: alanine transaminase
HCV: hepatitis C virus
HIV: human immunodeficiency virus
HTLV-1/2: human T-lymphotropic virus 1 and 2
IFN-γ: interferon-gamma
IL-10: interleukin-10
IL-12: interleukin-12
IL-17A: interleukin-17A
IL-1β: interleukin-1 beta
IL-2: interleukin-2
IL-4: interleukin-4
IL-6: interleukin-6
MHC-II: major histocompatibility complex II
NI: non-infected group
TNF-α: tumor necrosis factor alpha
Tregs: regulatory T-cells
