# Supplementary figures and images for: Liver and blood cytokine microenvironment in HCV patients is associated to liver fibrosis score: a proinflammatory cytokine ensemble orchestrated by TNF and tuned by IL-10
Source: BMC Microbiol. 2016 Jan 7;16:3. doi: 10.1186/s12866-015-0610-6 (PMC4705620; doi:10.1186/s12866-015-0610-6)

## Slide 1
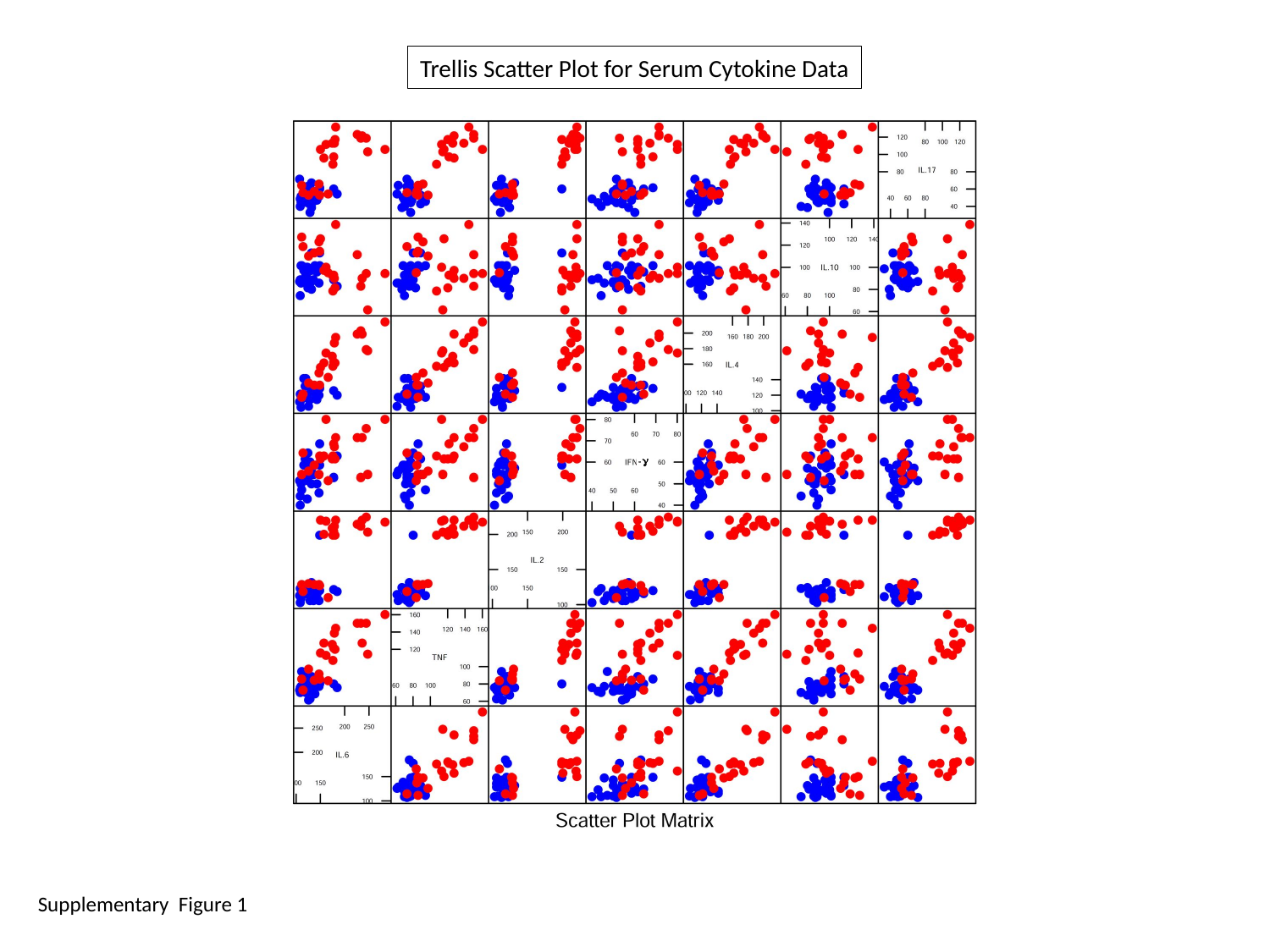

Trellis Scatter Plot for Serum Cytokine Data
Supplementary Figure 1

Supplement: Additional file 1: Figure S1. — Trellis scatter plot for serum cytokine analysis in HCV patients and non-infected controls. Scatter plot matrix analysis pointed out the relevant cytokine storm associated with HCV infection and reinforce the outstanding ability of serum cytokine attributes to cluster the HCV patients apart from non-infected individuals. (PPTX 2359 kb) [file 12866_2015_610_MOESM1_ESM.pptx]
